# Method for simulating dose reduction in digital mammography using the Anscombe transformation

**DOI:** 10.1118/1.4948502

**Published:** 2016-05-06

**Authors:** Lucas R. Borges, Helder C. R. de Oliveira, Polyana F. Nunes, Predrag R. Bakic, Andrew D. A. Maidment, Marcelo A. C. Vieira

**Affiliations:** Department of Electrical and Computer Engineering, São Carlos School of Engineering, University of São Paulo, 400 Trabalhador São-Carlense Avenue, São Carlos 13566-590, Brazil; Department of Radiology, Hospital of the University of Pennsylvania, University of Pennsylvania, 3400 Spruce Street, Philadelphia, Pennsylvania 19104; Department of Electrical and Computer Engineering, São Carlos School of Engineering, University of São Paulo, 400 Trabalhador São-Carlense Avenue, São Carlos 13566-590, Brazil

**Keywords:** dose reduction, digital mammography, Anscombe transformation, quantum noise

## Abstract

**Purpose:**

This work proposes an accurate method for simulating dose reduction in digital mammography starting from a clinical image acquired with a standard dose.

**Methods:**

The method developed in this work consists of scaling a mammogram acquired at the standard radiation dose and adding signal-dependent noise. The algorithm accounts for specific issues relevant in digital mammography images, such as anisotropic noise, spatial variations in pixel gain, and the effect of dose reduction on the detective quantum efficiency. The scaling process takes into account the linearity of the system and the offset of the detector elements. The inserted noise is obtained by acquiring images of a flat-field phantom at the standard radiation dose and at the simulated dose. Using the Anscombe transformation, a relationship is created between the calculated noise mask and the scaled image, resulting in a clinical mammogram with the same noise and gray level characteristics as an image acquired at the lower-radiation dose.

**Results:**

The performance of the proposed algorithm was validated using real images acquired with an anthropomorphic breast phantom at four different doses, with five exposures for each dose and 256 nonoverlapping ROIs extracted from each image and with uniform images. The authors simulated lower-dose images and compared these with the real images. The authors evaluated the similarity between the normalized noise power spectrum (NNPS) and power spectrum (PS) of simulated images and real images acquired with the same dose. The maximum relative error was less than 2.5% for every ROI. The added noise was also evaluated by measuring the local variance in the real and simulated images. The relative average error for the local variance was smaller than 1%.

**Conclusions:**

A new method is proposed for simulating dose reduction in clinical mammograms. In this method, the dependency between image noise and image signal is addressed using a novel application of the Anscombe transformation. NNPS, PS, and local noise metrics confirm that this method is capable of precisely simulating various dose reductions.

## 1. INTRODUCTION

To validate studies of radiation dose reduction, it is necessary to have a set of clinical images acquired from the same patient at different radiation levels. The availability of such images is limited, since these require repeated irradiation of patients. One way to overcome this limitation is to use realistic breast phantoms; anthropomorphic phantoms are capable of mimicking the appearance of breast tissues, either as physical models^1,2^ or through digital simulation.^3–5^

Another approach to validate dose reduction methods is to simulate the reduced dose by postprocessing clinical images.^6–12^ In fact, this method is used frequently when studying the influence of dose reduction on the detection of breast lesions.^13–15^ Saunders and Samei^6^ proposed a method to simulate different exposures that uses a noiseless image as input. However, it is not possible to acquire noiseless clinical images. Furthermore, the method is based on a radially symmetric noise power spectrum (NPS),^16^ which does not account for the anisotropic noise in digital mammograms.

Båth *et al.*^7^ presented a method capable of simulating dose reduction in radiographic images using information extracted from the 2D NPS, therefore accounting for the anisotropic behavior of the noise. However, the method is based on the assumption that the detective quantum efficiency (DQE)^16^ is approximately constant over the range of doses simulated. In digital mammography, the additive noise has a relevant influence on the DQE for low doses.^17^

Kroft *et al.*^8^ argued that to be clinically useful, the noise simulation algorithm has to be simple. With this argument, they proposed an alternative approach for measuring the amount of noise to be added to the image, discarding the NPS. This method was based on the local standard deviation of the image. However, as shown by the authors themselves,^9^ although the simulated images have the same local standard deviation as real images, they have different NPS. Since the standard deviation is not a good metric of the image noise when comparing images with different NPS,^18^ the clinical application of this method is questionable.

In recent work, Svalkvist and Båth^10^ presented a modification of the method proposed by Båth *et al.*^7^ that accounts for the DQE variation with dose. However, as pointed by the authors themselves,^10^ the method does not account for local variations in pixel gain, caused by the nonuniformities between detector elements.

Other methods have also been proposed recently,^11,12^ based on the NPS. However, to account for the spatial dependency of the variance, these methods propose the application of algorithms to remove flat-field corrections before simulating the dose reduction, to eliminate the spatial dependency of the noise.

A new method for simulating dose reduction in digital mammograms is proposed in this paper. The method is based on the local simulation of noise calculated using the local variance of uniform images. It can be applied to flat-fielded clinical images and accounts for the anisotropy of the noise and DQE variations. The method is based on a novel idea of inserting noise after applying a variance-stabilizing transformation (e.g., Anscombe transformation). In the variance-stabilized domain, noise is independent of the mean pixel value; therefore, the inserted noise can be modeled based only on the spatial dependency of the variance, thus accounting for the flat-field corrections. The dependency between the noise mask and the pixel value is created when the inverse transformation is applied.

## 2. BACKGROUND ON THE ANSCOMBE TRANSFORMATION

The Anscombe transformation is a variance-stabilizing transformation that converts a random variable with Poisson distribution into an approximately Gaussian distribution, with zero mean and unity variance.^19^ Let the degraded image, $g\left(x,y\right)$, at spatial coordinates *x* and *y*, be the random variable. The Anscombe transformation applied on $g\left(x,y\right)$ is given by the following:

$$A\left\{g\left(x,y\right)\right\}=2\sqrt{g\left(x,y\right)+\frac{3}{8}}.$$ (1)

The original inverse transformation proposed by the author^19^ is biased for small counting values $\left(\lambda <10\right)$; therefore in applications where the counting values are small, the results obtained after using the inverse transformation are different from the expected values. In recent work, Mäkitalo and Foi^20^ proposed a new approach for the inverse transformation, without any bias for small counts. Although counting values are considerably higher in mammography images $\left(\lambda \gg 10\right)$, in this work, we used the unbiased exact inverse, available online.^21^

**FIG. 1.**
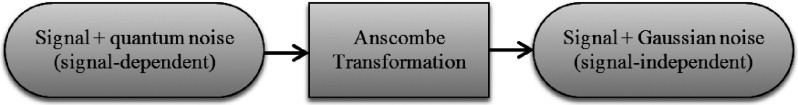
Schematic of a common application for the Anscombe transformation.

The Anscombe transformation has been commonly explored in the field of image denoising, where a noisy image *g*(*x*, *y*) is filtered in the Anscombe domain using filters designed to treat Gaussian signal-independent noise.^22,23^ Figure 1 illustrates the rationale for this transformation; the Anscombe transformation transforms the image noise into signal-independent noise.

**FIG. 2.**
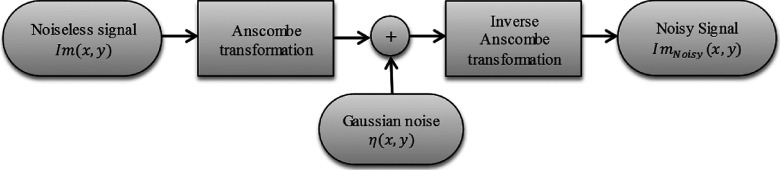
Novel application for the Anscombe transformation.

The new approach consists on adding signal-independent noise to the image in the Anscombe domain and applying the inverse transformation afterward. Figure 2 shows a schematic of the new application.

Let Im(*x*, *y*) be the noiseless original image and *η*(*x*, *y*) be a mask containing signal-independent noise. The image contaminated by signal-dependent noise, ${\text{Im}}_{\text{Noisy}}\left(x,y\right)$, is given by

$${\text{Im}}_{\text{Noisy}}\left(x,y\right)=A^{-1}\left\{A\left\{\text{Im}\left(x,y\right)\right\}+\eta \left(x,y\right)\right\}.$$ (2)

In the particular case when $\eta \left(x,y\right)$ is a Gaussian signal-independent noise mask with unity variance and zero mean, the inserted noise will follow the Poisson distribution.

**FIG. 3.**
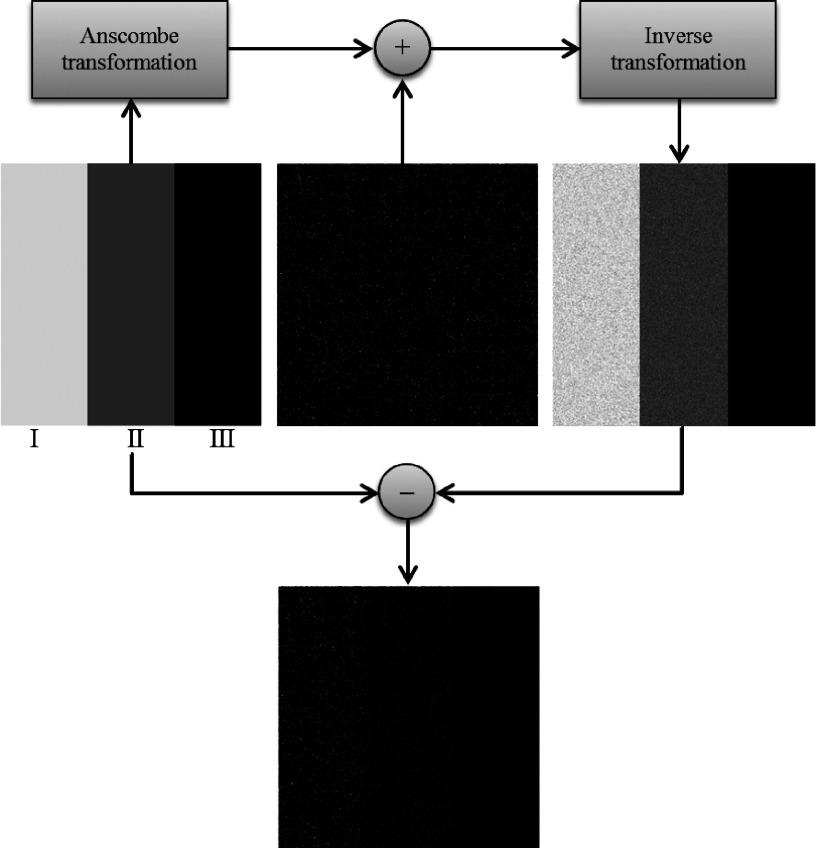
Top-left: synthetic noiseless image [Average signal: (I) 60 000 (II) 30 000 (III) 1000]. Top-center: Gaussian signal-independent noise (zero mean and unity variance). Top-right: noisy image after addition in the Anscombe domain and inverse Anscombe transformation. Bottom: Poisson signal-dependent noise subtracted from the noisy image. Contrast and brightness were improved for better visualization.

As a preliminary empirical test of this method, we created a 512 × 512 pixel synthetic image containing three distinct regions with various gray levels. Each region simulates a counting process with a different mean $\left(\lambda \right)$. The Anscombe transformation was applied to the synthetic image and a Gaussian signal-independent mask with zero mean and unity variance was added to the image in the Anscombe domain. After applying the inverse transformation proposed by Mäkitalo and Foi,^20^ a dependency was created between the noise mask and the signal. Figure 3 illustrates the results of this process. Note that after processing, the noise is signal-dependent.

Table I shows the standard deviation (std) from each simulated stripe from Fig. 3 (bottom), along with the average signal (*λ*) in that region and the expected theoretical standard deviation for a Poisson distribution, calculated^24^ using

$$\text{std}\left(X\right)=\sqrt{\lambda },$$ (3)

where *X* is a region of the image with average signal of *λ*.

Table I illustrates the potential of using the Anscombe transformation to contaminate a noiseless image with signal-dependent noise. However, three problems are associated with the use of this method for simulating noise in digital mammography images acquired with lower-radiation dose. First, a clinical mammogram must be used as input for the proposed method. However, such an image cannot be used as a noiseless approximation of the signal once it has been contaminated with noise from the acquisition system.

Second, the method produces unscaled pure Poisson noise. In clinical mammography, very few systems use photon-counting detectors; most systems use energy integrating detectors. Therefore, the noise added in the Anscombe domain should be different from a Gaussian with unity variance and must be estimated. Finally, the noise found in mammograms depends on the position of the pixel on the field, due to the flat-fielding process, which corrects the nonuniformity of the field caused by the heel effect and the oblique incidence of x-rays.^17^

In Sec. 3, we address each of these problems and present ways to solve them using the new algorithm proposed in this work.

## 3. MATERIALS AND METHODS

### 3.A. Method

Let *Y_o_*(*x*, *y*) be a clinical mammogram at coordinates *x* and *y*. To simulate a low-dose mammogram $\left(Y_{\text{sim}}\left(x,y\right)\right)$ acquired at the simulated dose *D*_sim_, it is necessary to acquire two uniform images [*H_o_*(*x*, *y*) and *H*_sim_(*x*, *y*)] at the original and simulated doses, respectively. These uniform images determine how much noise must be added to the clinical image acquired at the original dose to ensure that the noise is correctly simulated for the lower-dose acquisition.

The proposed method consists of three steps. In the first step, all of the images are linearized and the images acquired at the original dose are scaled by the dose reduction factor. The second step is to simulate the noise distribution at each pixel of the image, using local information extracted from the uniform images acquired at the different doses. Lastly, a dependency is created between the simulated noise and the clinical image acquired using the standard radiation dose to generate the simulated low dose image.

**FIG. 4.**
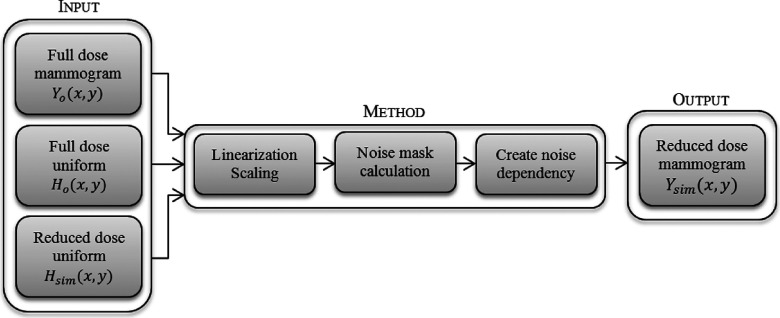
Overview of the method proposed in this work.

This processing method assumes that the input images are in the raw format. The raw format, identified as “for processing” in the DICOM header of the image, contains image data which is minimally processed, lacking processing to improve tissue contrast for easier interpretation by the radiologist. Figure 4 shows an overview of the proposed method.

Section 3.A is divided into three subsections where each step of the algorithm will be explained in more detail. Indexes *L*, *S*, and *A* indicate that the associated variables are linearized, scaled, and in the Anscombe domain, respectively.

#### 3.A.1. Linearization and scaling

Images acquired with lower-radiation dose have lower overall signal, as compared to standard-dose mammograms. Therefore, initially we need to adjust the gray level of the clinical mammogram to match the simulated lower-dose acquisition. If we consider an x-ray system where the radiation dose is the input variable and the mean pixel value is the output, it is possible to scale the gray level of an image acquired by this equipment as long as the relation between these quantities is linear.

Although the relationship between mean pixel value and radiation dose usually has an offset, it is possible to remove this value.^7^ One simple way to correct this problem is to subtract the offset from the image before the processing is performed, and then to add the offset back after all the processing steps are complete. To find the offset, we acquire at least two uniform images at different radiation doses using the equipment to be simulated. Then, we calculate a linear regression to find the relation between dose and mean pixel value of the uniform images. The constant term of the regression is the photodetector offset.

After linearizing the image, it is possible to scale the gray levels to simulate the distribution of an image acquired at a lower-radiation dose. Thus, pixel is multiplied by a constant term, hereby denoted the reduction rate $\left(\alpha \right)$. This constant can be calculated as the ratio between the current-exposure time product of the full dose mammogram and the current-exposure time product of the reduced-dose mammogram. Therefore, the linearized scaled image (*Y*^*L*,*S*^(*x*, *y*)) is given by

$$Y^{L,S}\left(x,y\right)=\alpha Y^{L}\left(x,y\right).$$ (4)

#### 3.A.2. Noise calculation

Since clinical mammograms intrinsically contain noise, it is vital to have information about the amount of noise present in an image acquired by the particular system that will be simulated. Using a uniform image exposed with the same parameters as the clinical image, it is possible to obtain a good approximation of the noise present in the clinical exam. The second challenge is to measure the noise present in the low dose configuration. Again, this information can be extracted from a uniform image acquired at the simulated radiation dose. Due to the flat-fielding process, the noise in a mammogram is also a function of the spatial position in the detector array. Thus, the noise must either be simulated locally or the flat-field correction must be removed. In this work, we propose a local method, which allows the simulation of flat-fielded images.

Traditionally, the expected local standard deviation of the noise to be added to the original image is calculated as follows:

$$\sigma_{\text{sim}}\left(x,y\right)=\sqrt{\sigma_{H_{\text{sim}}^{L}}^{2}\left(x,y\right)-\sigma_{H_{o}^{L,S}}^{2}\left(x,y\right)},$$ (5)

where *σ*_sim_(*x*, *y*) is the expected local standard deviation of the noise, $\sigma_{H_{\text{sim}}^{L}}^{2}\left(x,y\right)$ and $\sigma_{H_{o}^{L,S}}^{2}\left(x,y\right)$ are the variance masks calculated locally using a square window that runs through $H_{\text{sim}}^{L}$ and $H_{o}^{L,S}$, respectively. To create the noise mask, an array of randomly generated Gaussian noise with zero mean and unity variance, the same size as the clinical image, is multiplied by the standard deviation calculated using Eq. (5). Next, the noise mask is added to the scaled clinical mammogram $Y_{o}^{L,S}$.

There are two problems associated with this simple way to incorporate the noise. First, the quantum noise present in digital mammograms is signal-dependent, i.e., its local variance depends on the local mean of the signal. The noise calculated above is not dependent on the signal found in the clinical image, thus a dependency must be created before adding them together. Second, quantum noise cannot be described as a uniform additive fraction of the signal.

**FIG. 5.**
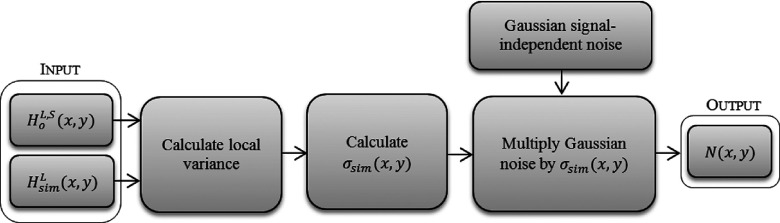
Overview of the noise creation process where $H_{o}^{L,S}\left(x,y\right)$ is the uniform image acquired with full dose after linearization and scaling; $H_{\text{sim}}^{L}\left(x,y\right)$ is the uniform image acquired with the simulated dose after linearization and *σ*_sim_(*x*, *y*) is the standard deviation mask as calculated from Eq. (5). $N\left(x,y\right)$ is the noise mask with the local variance modulated by $\sigma_{\text{sim}}\left(x,y\right)$.

The following is a new approach for creating signal-dependent noise that is additive to the signal. Figure 5 shows an overview of this noise creation process, in which the Anscombe transformation is used to incorporate the calculated noise to the clinical image.

#### 3.A.3. Signal-dependency and additivity

When the Anscombe transformation is applied to a signal, Poisson noise is converted to additive signal-independent noise.^19^ Therefore, once in the Anscombe domain, the noise mask can be correctly added to the signal. After applying the inverse transformation, the dependency between the noise mask and the signal is also created and the resulting image is similar to a low-dose mammogram.

The first step to add the noise mask and create the dependency between it and the scaled clinical mammogram is to apply the Anscombe transformation to both images, using Eq. (1). However, the Anscombe transformation must be applied to a signal contaminated with noise, and the noise mask calculated previously does not contain any signal.^19^ Therefore, to allow the correct use of the Anscombe transformation, a positive DC signal has to be added to the noise mask prior to the application of the transformation. The added signal is the mean pixel value of the uniform image acquired at the simulated dose $\left(H_{\text{sim}}^{L}\left(x,y\right)\right)$. Once in the Anscombe domain, it is necessary to subtract the DC component to get the noise mask. Equation (6) presents the mathematical expression for the process,

$$N^{A}\left(x,y\right)=A\left\{N\left(x,y\right)+\bar{H_{\text{sim}}^{L}}\right\}-A\left\{\bar{H_{\text{sim}}^{L}}\right\},$$ (6)

where $\bar{H_{\text{sim}}^{L}}$ is the mean pixel value of the linearized uniform image acquired at the simulated dose, *N*(*x*, *y*) is the noise mask calculated as shown in Fig. 5, and $N^{A}\left(x,y\right)$ is the noise mask in the Anscombe domain.

**FIG. 6.**
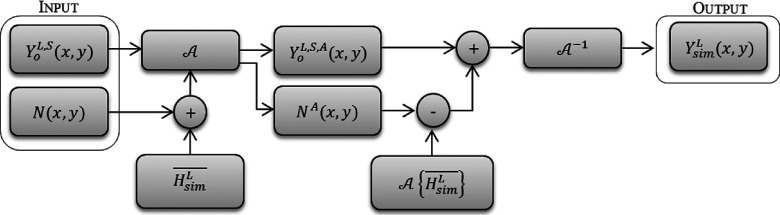
Novel method for creating dependency between noise and signal, where $Y_{o}^{L,S}\left(x,y\right)$ is the clinical mammogram acquired with full dose after linearization and scaling process, *N*(*x*, *y*) is the noise mask calculated previously, $\bar{H_{\text{sim}}^{L}}$ is the mean pixel value of the uniform image acquired with the simulated dose.

Next, the noise mask and the linearized scaled clinical image can be added together to generate an image with additive Gaussian noise in the Anscombe domain. This image approximates the image obtained if the Anscombe transformation was applied to a mammographic image acquired with lower-radiation dose. The next step for our method is to apply the inverse Anscombe transformation to that image in order to obtain the simulated image in the spatial domain. Figure 6 shows an overview of the process.

Finally, the last step of the method is to add the offset back to the image, to guarantee the same behavior as a clinical image, as follows:

$$Y_{\text{sim}}\left(x,y\right)=Y_{\text{sim}}^{L}\left(x,y\right)+\theta .$$ (7)

### 3.B. Materials

#### 3.B.1. Images

To assess the performance of the simulation method proposed in this work, a set of FFDM images was acquired using an anthropomorphic breast phantom, prototyped by CIRS, Inc. (Reston, VA) and the University of Pennsylvania.^25^ Four different technique factors, resulting in four different entrance doses to the phantom (6.05, 5.29, 4.53, and 3.02 mGy), were used to validate the simulation method.

A few reasons are presented to justify why a physical phantom was chosen to validate this work. First, the physical phantom allows repeated exposures at different radiation levels without putting a patient’s health at risk due to radiation exposure. Second, by using the physical phantom properly affixed to the breast support, it is possible to avoid any motion throughout the experiment, obviating the need for image registration. Finally, physical phantoms are subjected to an actual x-ray exposure, ensuring that every noise characteristic found in the clinical situation is found in the image of the physical phantom. By comparison, in digital phantoms, the exposure process is simulated using a mathematical model; therefore the noise behavior is simulated and might have slight differences when compared to a clinical exposure.

**FIG. 7.**
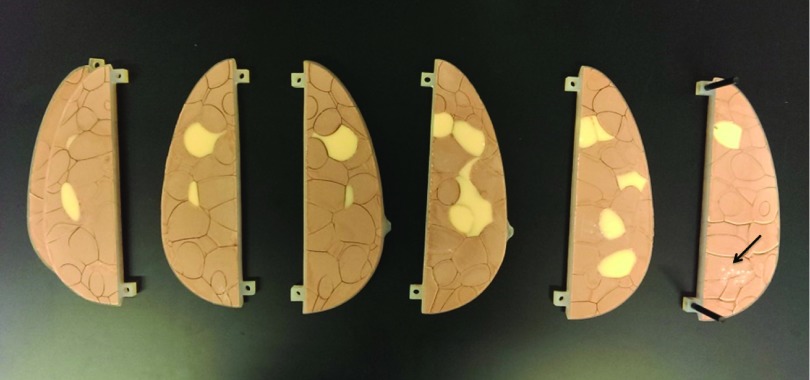
Slabs of the physical phantom used in this work. The arrow indicates one of the inserted microcalcification clusters.

The breast phantom consists of six slabs, each containing simulated anatomical structures manufactured using tissue mimicking materials, based upon a realization of the companion breast software phantom.^3^ The phantom simulates a 450 ml breast, compressed to 5 cm, with 17% volumetric breast density (excluding the skin). In addition to the normal breast anatomy, individual pieces of calcium oxalate (99%, Alfa Aesar, Ward Hill, MA) with different sizes were placed between slabs of the phantom to mimic a cluster of microcalcifications. Figure 7 shows a photograph of all slabs of the anthropomorphic breast phantom used in this study.

**FIG. 8.**
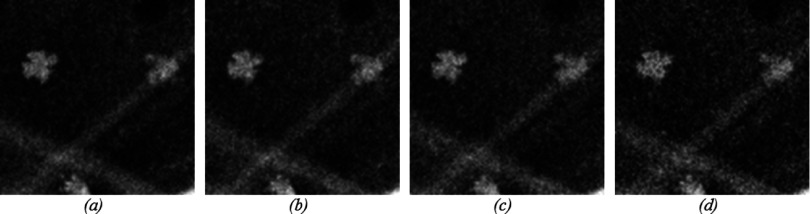
Magnified region of the phantom containing a cluster of microcalcifications. (a) 6.05 mGy, (b) 5.29 mGy, (c) 4.53 mGy, and (d) 3.02 mGy.

A set of phantom images was acquired using a clinical mammography imaging system (Selenia Dimensions, Hologic, Bedford, MA) at the hospital of the University of Pennsylvania. First, we acquired one FFDM image of the phantom using the automatic exposure control (AEC) mode of the clinical machine. Then, we switched to the manual mode and acquired four sets of images, containing five images each, using the same kVp and target/filter combination as provided by the AEC mode but changing the exposure time in steps ranging from the original value (standard dose) to half of the standard dose. Each of these sets had a different current-exposure time resulting in different values of entrance dose to the phantom: 6.05, 5.29, 4.53, and 3.02 mGy. These doses correspond to 100%, 87.5%, 75%, and 50% of the standard dose provided by the AEC mode for this particular breast phantom. All images were acquired using the antiscatter grid. Figure 8 shows one magnified region containing a cluster of microcalcifications at each exposure configuration used in this work.

Each exposure configuration was repeated with a uniform phantom, i.e., a 4 cm thick acrylic block commonly used for flat-fielding the mammography system. Two uniform images were acquired for each combination of exposure parameters.

#### 3.B.2. Metrics

The metrics used to compare the real and simulated images were chosen taking into account two characteristics of the image: spatial distribution and power spectrum (PS). Since noise depends on the position of the pixel in the field, these metrics were calculated locally inside a 14.3 × 3.8 cm (2048 × 512 pixels) ROI containing the breast to avoid false statistics from the background. A regular nonoverlapping square mask with 64 × 64 pixels (0.45 × 0.45 cm) was used to calculate the variance from both simulated and real images. The center of the mask was shifted by 64 pixels before calculating each value; therefore, the total number of samples was 256. Each point was plotted in a graph to allow visual comparison of the results. Relative error was calculated to quantify similarity between images and the average difference was reported along with the 95% confidence interval (C.I.).

The normalized noise power spectrum (NNPS) was used to analyze noise in the frequency domain. This metric is a normalized form of the noise power spectrum, defined as follows:^16^

$$\text{NPS}\left(u,v\right)=\underset{\underset{x}{N},N_{y},M\to \infty }{\lim}\left(\frac{N_{x}N_{y}\Delta x\Delta y}{M}\right)\overset{M}{\underset{m=1}{\sum }}{\left|F\left\{I_{m}\left(x,y\right)-S_{m}\left(x,y\right)\right\}\right|}^{2},$$ (8)

where *N_x_* and *N_y_* are the ROI dimensions, Δ*x* and Δ*y* are the pixel dimensions in the *x* and *y* directions, respectively, *S*(*x*, *y*) is an approximation of the noiseless signal, *M* is the total number of ROI’s used, and F indicates the Fourier transform. The normalization is performed by dividing the spectra by the square of the large area signal, as defined by the following:^16^

$$\mathrm{NNPS}\left(u,v\right)=\frac{\mathrm{NPS}\left(u,v\right)}{L^{2}},$$ (9)

where *L* is the large area signal of the region. The NNPS was calculated using uniform images, where there is no information about the breast texture complexity. We also calculated the normalized PS.

The 1D spectrum was calculated^16^ and plotted for each dose reduction to allow visual evaluation of the similarity between simulated and real images. The mean and the standard deviation of the difference between the spectra of the simulated and real images were calculated. This calculation was restricted to 1.5–7.1 mm^−1^, where the spectra did not show strong frequency dependence. The average of the difference was calculated and presented along with the 95% confidence interval. Relative error was also reported.

**FIG. 9.**
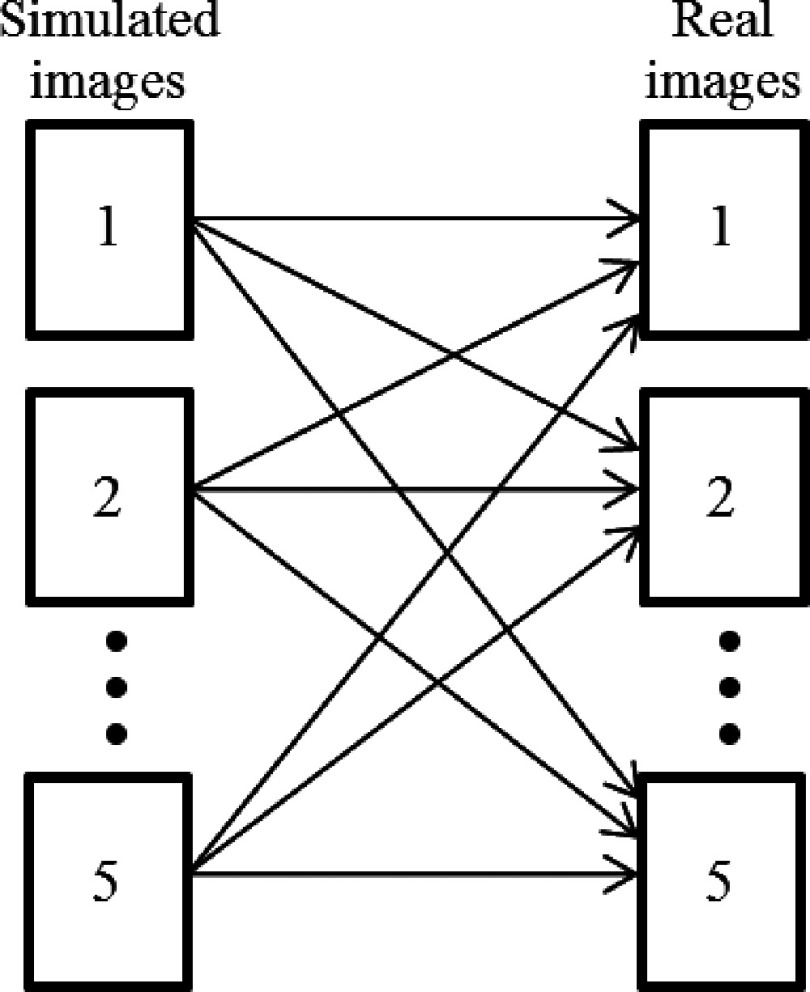
Method used for crossed comparison between simulated and real images.

Comparisons between simulated and real image sets were performed using the method shown in Fig. 9 in which each simulated image is compared to each real image, generating more statistically relevant data.

## 4. RESULTS

### 4.A. Preliminary noise analysis

**FIG. 10.**
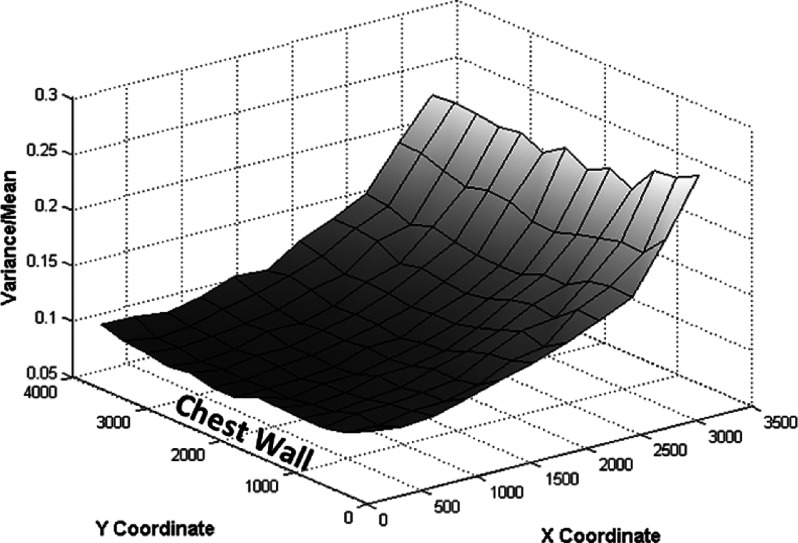
Relation between variance and mean throughout the x-ray field.

Preliminary investigation of the noise was performed using uniform images. The graph shown in Fig. 10 shows the ratio between local noise variance and local mean pixel value.

Equation (3) indicates that a uniform image contaminated by Poisson noise would have a constant relation equal to one. However, in Fig. 10, the ratio between noise variance and mean pixel value ranges from 0.1 to 0.3, depending on the position in the field. This spatial dependence is a result of the flat-field correction performed during the system calibration, which corrects for the heel effect and x-ray oblique incidence. Figure 10 shows the importance of using a dose reduction method that is locally adaptive.

### 4.B. Photodetector offset

The first step in evaluating the new method was calculating the photodetector offset. To calculate the value, four different doses were used and the relation between mean pixel value and entrance dose to the phantom was represented by linear regression. The uniform images were acquired imaging a 4 cm uniform PMMA block at four different exposure configurations, all of them acquired with 29 kVp, with tungsten anode filtered with rhodium. The current–time parameter was initially set to 160 mAs, followed by 140, 120, and 80 mAs. Two images were acquired at each dose.

**FIG. 11.**
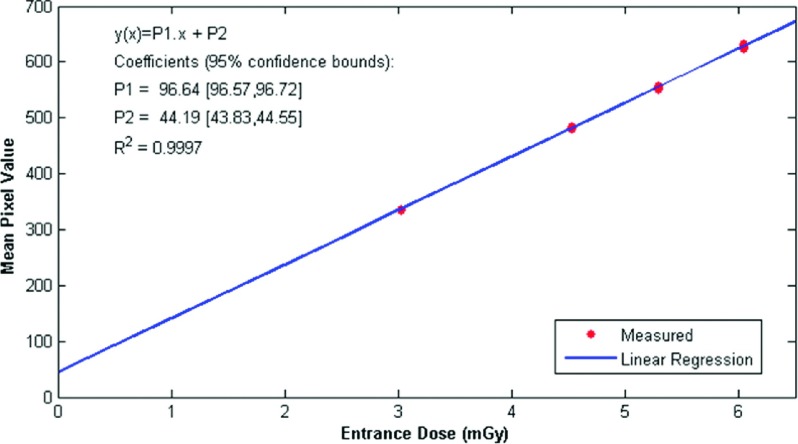
Linear regression of the relation between mean pixel value and entrance dose to the phantom. The linear coefficient is important for the linearization process.

To calculate the linear regression, the mean pixel value was calculated in 207 different regions of the uniform images using a 64 × 64 pixels window, totaling 1656 measured points. Figure 11 shows the results.

The offset for the machine used in this study is approximately 44. Although the manufacturer of the mammographic unit estimates the offset to be 50,^26^ this value was measured experimentally to guarantee the correct linearity of the images, as in our experience, this value can encompass small nonlinearities found with various imaging systems.

### 4.C. Method evaluation

#### 4.C.1. Physical phantom

**FIG. 12.**
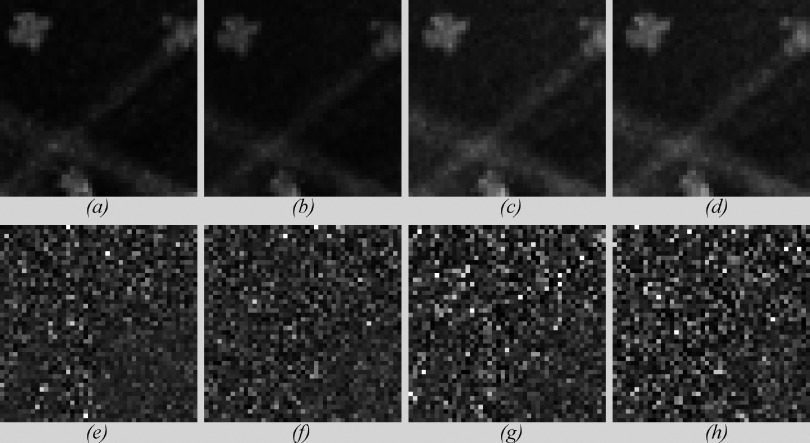
Top-row: magnified view of a region of interest extracted from (a) real image acquired with 5.29 mGy. (b) Simulated image (5.29 mGy). (c) Real image acquired with 3.02 mGy. (d) Simulated image (3.02 mGy). Bottom-row: residual noise calculated for (e) real image acquired with 5.29 mGy. (f) Simulated image (5.29 mGy). (g) Real image acquired with 3.02 mGy. (h) Simulated image (3.02 mGy).

Examples of simulated and real images of the physical phantom are shown in Fig. 12. It shows a magnified view of a region of interest extracted from real and simulated images at two radiation doses: 5.29 and 3.02 mGy. The magnified view allows better visualization of the noise in each image. Along with the ROIs are the residual noise masks calculated by subtracting the noiseless approximation of the signal from each image. The “noiseless” signal was approximated by averaging five realizations for each dose.

Visual analysis of Fig. 12 provides evidence that the simulated and real images are very similar. However, it is important to use appropriate metrics to evaluate the proposed method objectively. Local metrics such as variance are not a good measurement of similarity when comparing x-ray images with distinct noise power spectra.^18^ Thus, NNPS and PS are used to evaluate the simulation method.

**FIG. 13.**
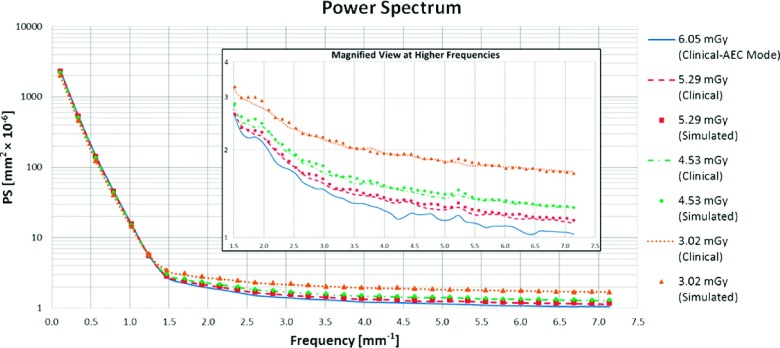
Normalized PS for real and simulated images acquired with different radiation doses. In detail: magnified view of the high frequencies of the spectrum.

Figure 13 provides a comparison between PS at different doses for clinical and simulated images. We performed crossed comparisons between real and simulated images. In Table II, values are reported after calculating the absolute percent error between the simulated and real images at each frequency and averaging them. In addition, it shows the average difference between simulated and real PS and its 95% confidence interval. For the PS, a regular nonoverlapping 0.90 × 0.90 cm window was used, resulting in 1600 comparisons per dose, 4800 in total.

The averaged error for the PS is less than 2.5%. The confidence interval of the average difference does not span zero, indicating that there is a bias in the PS; however, the average difference is 100 times smaller than the average signal, showing that the bias is small.

**FIG. 14.**
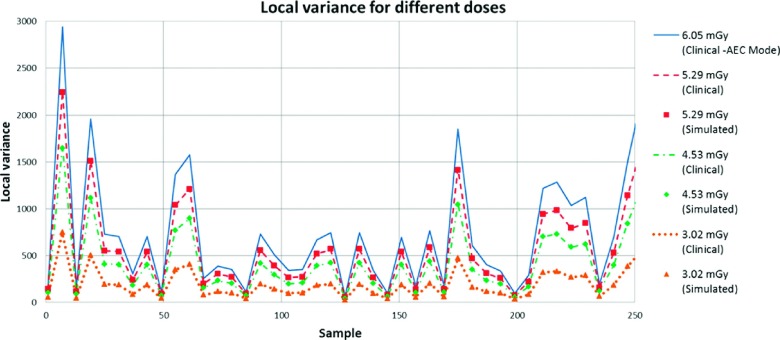
Local variance for real and simulated images acquired with different radiation dose.

Using the same 0.45 × 0.45 cm window, we calculated the local variance from both simulated and real images and plotted the different regions in the graph shown in Fig. 14 (as explained in Sec. 3.B.2). Similarly to the previous image, in Fig. 14 lines represent clinical images acquired at different doses while markers identify simulated images. Each point is the average of five different realizations.

Again, we performed crossed comparison between clinical and simulated images. A total of 19 400 comparisons were averaged and reported at Table II, along with the average difference and the 95% confidence interval. It is important to note that averaged errors are less than 1.0% for the local variance and the confidence interval of the average difference spans zero, indicating a high correlation between simulated and real variance.

#### 4.C.2. Uniform images

**FIG. 15.**
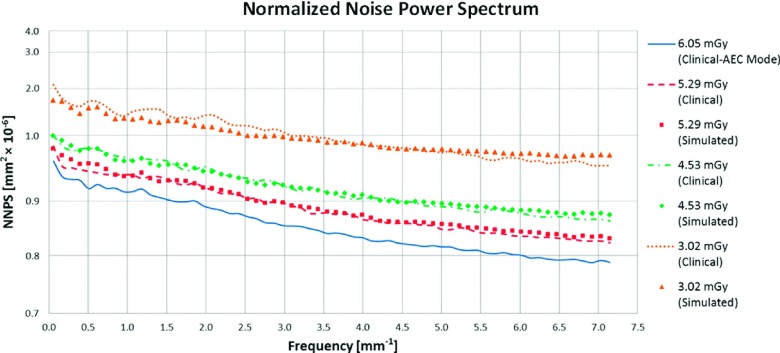
Normalized noise power spectrum calculated for real and simulated uniform images at different radiation doses.

An additional study was performed using uniform images. In this section, we present the results of the simulation method applied to these uniform images, acquired using the radiograph factors described in Sec. 4.B. This analysis is important to show that the low frequency components of the spectrum are not biased by the DC component of the signal. Two images were acquired at each dose and the results of the simulation were compared using crossed comparison as presented in Sec. 3.B.2 for the physical phantom images. Figure 15 shows the NNPS of clinical and simulated uniform images at different radiation doses.

**FIG. 16.**
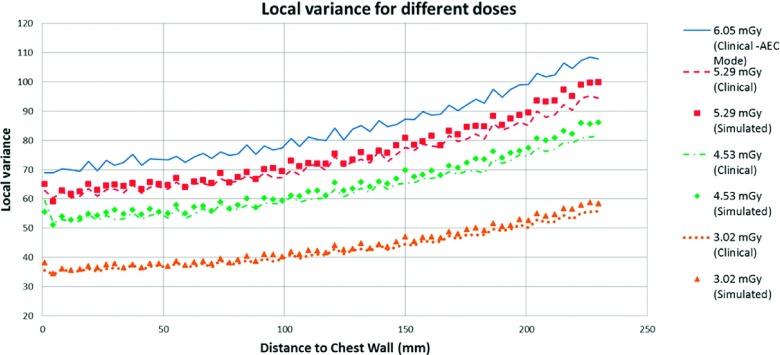
Local variance calculated for real and simulated uniform images at different radiation doses.

The noise insertion resulted in visually similar NNPS for each simulated dose, as seen in Fig. 15. The second metric used to compare the uniform images was the local variance. Figure 16 shows the local variance calculated using 64 × 64 pixels (0.45 × 0.45 cm) non-overlapping windows located at different positions of the field.

As expected, the noise variance increases in regions further from the chest wall. Figure 16 indicates that the simulation method was capable of approximating the local variance of a real acquisition.

Table III shows the absolute relative error calculated for the NNPS and local variance at each radiation dose when comparing simulated and real uniform images. In addition, it shows the average difference between simulated and real noise power spectra, along with the 95% C.I.

The reported averaged error was maintained lower than 3.8% for the NNPS and lower than 3.0% for the local variance, which indicates that the simulation method is capable of approximating the characteristics of real acquisitions even in uniform images, where it is possible to have a good approximation of the added noise. The confidence interval of the average difference does not span zero, indicating that there is a bias on the noise simulation. However, the values are insignificant when compared to the average signal.

## 5. DISCUSSION

A new method for simulating dose reduction in flat-fielded digital mammograms is proposed in this paper. The method is based on the local simulation of noise calculated using the local variance of uniform images. It can be applied to flat-fielded clinical images and accounts for the anisotropy of the noise and DQE variations. The method is based on a novel idea of inserting noise after applying a variance-stabilizing transformation (e.g., Anscombe transformation). In the variance stabilized domain, noise is independent of the mean pixel value; therefore the inserted noise mask can be modeled based only on the spatial dependency of the variance, thus accounting for the flat-field correction. In this way, the dependency between the noise mask and the pixel value is created when the inverse transformation is applied.

The proposed method accounts for the anisotropic behavior of the noise found in digital mammograms, since it uses 2D uniform images for measuring quantum noise. Also, it accounts for noise present in the input mammogram by measuring the local variance of a uniform image acquired at the same radiographic technique. No assumptions regarding the dependency between radiation dose and DQE are necessary, since noise is measured at the actual simulated dose. Furthermore, the method is capable of simulating noise locally, taking into account the spatial dependency between pixel value and variance along the field, caused by the digital mammography acquisition system. Such information is lost when only the NPS is used.

Preliminary analysis of the noise extracted from uniform images supported the development of the local simulation method proposed in this work. It is apparent from Fig. 10 that the quotients of the variance and mean pixel value have different values throughout the field; this is attributable to the pixel gain calibration that occurs during the acquisition process and the flat-fielding. As discussed above, the simple insertion of Poisson noise into clinical images does not accurately simulate dose reduction.

It is important to note that, although the NPS is not used explicitly by the proposed method when generating the noise mask, the noise mask image is real and hence intrinsically contains noise with the correct NPS. The graph reported in Fig. 14 supports the visual verification of the correspondence between clinical and simulated images in terms of local variance. In addition, it is noticeable that lower levels of radiation produced lower local variance, which is consistent with the expected behavior from an image contaminated by quantum noise.

Some limitations and future work are now addressed. The current version of the simulation method was validated assuming that dose reduction is performed exclusively by a decrease of the current-exposure time product (mAs), following the majority of previous methods.^6–10^ Future work should include analysis of simulating changes to other parameters such as tube peak voltage (kVp) and the target/filter combination, as well as greater reduction in dose, such as that seen in creating tomosynthesis projection images.

The validation of this method was performed using images acquired with a-Se detectors. The validity of such method has to be tested before being applied to other detectors, such as CsI/TFT, which have significant correlated noise. The method was tested using a phantom equivalent to a 5 cm breast using a 4 cm uniform PMMA block. Further studies must be performed to analyze the influence that the thickness of the PMMA block might exert on the simulation accuracy.

Another potential application for this method is simulating various radiation dose levels in virtual clinical trials (VCTs). Further studies are needed to separate and characterize the different components of the noise; in this way, it is possible to simulate the various sources of noise separately.

## 6. CONCLUSION

To the best of our knowledge, this work presents the first noise insertion method for simulating dose reduction to be performed in a variance-stabilizing domain (e.g., Anscombe domain). The method developed allows the modeled noise to account for the spatial dependency of the noise, allowing it to be applied to flat-fielded images.

## Figures and Tables

**TABLE I.** Comparison between theoretical std and measured std obtained using the inverse Anscombe transformation to convert Gaussian signal-independent noise into Poisson signal-dependent noise.

Region	Average signal $\left(\lambda \right)$ (from Fig. 3, top-left)	Measured std (from Fig. 3, bottom)	Expected std [from Eq. (3)]
I	60 000	245.4	245.0
II	30 000	173.0	173.2
III	1 000	31.6	31.6

**TABLE II.** Comparison between PS and variance for the simulated and real images acquired at three different doses, average difference between variables, and 95% C.I. of the difference.

Radiation dose	Calculated metrics	Average error (%)	Average difference [95% C.I.]
5.29 mGy	PS	2.47 ± 1.78	0.28 [0.26 0.29] × 10^−7^
	Variance	0.81 ± 0.66	0.28 [−1.01 1.52]
4.53 mGy	PS	2.41 ± 1.88	0.32 [0.24 0.40] × 10^−7^
	Variance	0.85 ± 0.70	1.90 [−1.02 2.57]
3.02 mGy	PS	2.15 ± 1.69	0.19 [0.06 0.32] × 10^−7^
	Variance	1.00 ± 0.84	0.91 [−0.71 2.93]

**TABLE III.** Comparison between NNPS and variance for the simulated and real uniform images acquired at 3 different doses, average difference between variables and 95% C.I. of the difference.

Radiation dose	Calculated metrics	Average error (%)	Average difference [95% C.I.]
5.29 mGy	NNPS	2.61 ± 0.75	0.20 [0.13 0.27] × 10^−7^
	Variance	2.78 ± 0.24	2.06 [2.44 1.68]
4.53 mGy	NNPS	2.35 ± 0.50	0.07 [0.00 0.15] × 10^−7^
	Variance	2.83 ± 0.33	1.91 [2.29 1.54]
3.02 mGy	NNPS	3.79 ± 0.25	0.27 [0.05 0.48] × 10^−7^
	Variance	2.94 ± 0.25	1.37 [1.55 1.18]

## References

[1] A.-K. Carton, P. Bakic, C. Ullberg, H. Derand, and A. D. A. Maidment, “Development of a physical 3D anthropomorphic breast phantom,” Med. Phys. 38(2), 891–896 (2011). 10.1118/1.3533896 21452726 PMC4108620

[2] A. Nolte, N. Kiarashi, E. Samei, W. P. Segars, and J. Y. Lo, “A second generation of physical anthropomorphic 3D breast phantoms based on human subject data,” Proc. SPIE 9033, 90331Y (2014). 10.1117/12.2043703

[3] D. D. Pokrajac, A. D. A. Maidment, and P. R. Bakic, “Optimized generation of high resolution breast anthropomorphic software phantoms,” Med. Phys. 39(4), 2290–2301 (2012). 10.1118/1.3697523 22482649 PMC3337667

[4] K. Bliznakova, S. Suryanarayanan, A. Karellas, and N. Pallikarakis, “Evaluation of an improved algorithm for producing realistic 3D breast software phantoms: Application for mammography,” Med. Phys. 37(11), 5604–5617 (2010). 10.1118/1.3491812 21158272 PMC2967417

[5] N. Kiarashi, J. Y. Lo, Y. Lin, L. C. Ikejimba, S. V. Ghate, L. W. Nolte, J. T. Dobbins 3rd, W. P. Segars, and E. Samei, “Development and application of a suite of 4-D virtual breast phantoms for optimization and evaluation of breast imaging systems,” IEEE Trans. Med. Imaging 33(7), 1401–1409 (2014). 10.1109/TMI.2014.2312733 24691118 PMC4226410

[6] R. S. Saunders and E. Samei, “A method for modifying the image quality parameters of digital radiographic images,” Med. Phys. 30(11), 3006–3017 (2003). 10.1118/1.1621870 14655948

[7] M. Båth, M. Håkansson, A. Tingberg, and L. G. Månsson, “Method of simulating dose reduction for digital radiographic systems,” Radiat. Prot. Dosim. 114(1-3), 253–259 (2005). 10.1093/rpd/nch540 15933117

[8] L. J. M. Kroft, W. J. H. Veldkamp, B. J. A. Mertens, J. P. A. Van Delft, and J. Geleijns, “Detection of simulated nodules on clinical radiographs: Dose reduction at digital posteroanterior chest radiography,” Radiology 241(2), 392–398 (2006). 10.1148/radiol.2412051326 17057066

[9] W. J. H. Veldkamp, L. J. M. Kroft, J. P. A. Van Delft, and J. Geleijns, “A technique for simulating the effect of dose reduction on image quality in digital chest radiography,” J. Digital Imaging 22(2), 114–125 (2009). 10.1007/s10278-008-9104-5 PMC3043684 18259814

[10] A. Svalkvist and M. Båth, “Simulation of dose reduction in tomosynthesis,” Med. Phys. 37(1), 258–269 (2010). 10.1118/1.3273064 20175489

[11] A. Mackenzie, D. R. Dance, A. Workman, M. Yip, K. Wells, and K. C. Young, “Conversion of mammographic images to appear with the noise and sharpness characteristics of a different detector and x-ray system,” Med. Phys. 39(5), 2721–2734 (2012). 10.1118/1.4704525 22559643

[12] A. Mackenzie, D. R. Dance, O. Diaz, and K. C. Young, “Image simulation and a model of noise power spectra across a range of mammographic beam qualities,” Med. Phys. 41(12), 121901 (14pp.) (2014). 10.1118/1.4900819 25471961

[13] P. Timberg, M. Ruschin, M. Bath, B. Hemdal, I. Andersson, S. Mattsson, D. Chakraborty, R. Saunders, E. Samei, and A. Tingberg, “Potential for lower absorbed dose in digital mammography: A JAFROC experiment using clinical hybrid images with simulated dose reduction,” Proc. SPIE 6146, 614614 (2006). 10.1117/12.653419

[14] R. S. Saunders, J. A. Baker, D. M. Delong, J. P. Johnson, and E. Samei, “Does image quality matter? Impact of resolution and noise on mammographic task performance,” Med. Phys. 34(10), 3971–3981 (2007). 10.1118/1.2776253 17985642

[15] M. Ruschin, P. Timberg, M. Bath, B. Hemdal, T. Svahn, R. Saunders, E. Samei, I. Andersson, S. Mattsson, D. P. Chakraborty, and A. Tingberg, “Dose dependence of mass and microcalcification detection in digital mammography: Free response human observer studies,” Med. Phys. 34(2), 400–407 (2007). 10.1118/1.2405324 17388156 PMC1892618

[16] I. A. Cunningham, “Applied linear-systems theory,” in Handbook of Medical Imaging: Volume 1 Physics Psychophysics, edited by Beutel J., Kundel H. L., and Metter R. L. V. (SPIE, Bellingham, 2000), Chap. 2, pp. 79–159.

[17] M. Yaffe, “Digital mammography,” in Handbook of Medical Imaging: Volume 1 Physics Psychophysics, edited by Beutel J., Kundel H. L., and Metter R. L. V. (SPIE, Bellingham, 2000), Chap. 5, pp. 329–372.

[18] A. E. Burgess, “The Rose model, revisited,” J. Opt. Soc. Am. A 16(3), 633–646 (1999). 10.1364/JOSAA.16.000633 10069050

[19] F. J. Anscombe, “The transformation of Poisson, binomial and negative-binomial data,” Biometrika 35(3-4), 246–254 (1948). 10.1093/biomet/35.3-4.246

[20] M. Mäkitalo and A. Foi, “Optimal inversion of the Anscombe transformation in low-count Poisson image denoising,” IEEE Trans. Image Process. 20(1), 99–109 (2011). 10.1109/tip.2010.2056693 20615809

[21] M. Mäkitalo and A. Foi, “Optimal inversion of the Anscombe and generalized Anscombe variance-stabilizing transformations,” 2015, available at http://www.cs.tut.fi/~foi/invansc/#ref~software.

[22] L. C. S. Romualdo, M. A. C. Vieira, H. Schiabel, N. D. A. Mascarenhas, and L. R. Borges, “Mammographic image denoising and enhancement using the Anscombe transformation, adaptive Wiener filtering, and the modulation transfer function,” J. Digital Imaging 26(2), 183–197 (2013). 10.1007/s10278-012-9507-1 PMC3597965 22806627

[23] N. D. A. Mascarenhas, C. A. N. Santos, and P. E. Cruvinel, “Transmission tomography under Poisson noise using the Anscombe transformation and Wiener filtering of the projections,” Nucl. Instrum. Methods Phys. Res., Sect. A 423(2), 265–271 (1999). 10.1016/S0168-9002(98)00925-5

[24] F. A. Haight, “Handbook of the Poisson distribution,” J. Oper. Res. Soc. 18(4), 478–479 (1967). 10.2307/3007702

[25] L. Cockmartin, P. R. Bakic, H. Bosmans, A. D. A. Maidment, H. Gall, M. Zerhouni, and N. W. Marshall, “Power spectrum analysis of an anthropomorphic breast phantom compared to patient data in 2D digital mammography and breast tomosynthesis,” in *Proceedings of IWDM* (Springer International Publishing, Gifu City, Japan, 2014), Vol. 8539, pp. 423–429.

[26] K. C. Young and J. M. Oduko, “Technical evaluation of the Hologic Selenia full field digital mammography system with a tungsten tube,” NHSBSP Equipment Report 0801, 2008.

